# Philadelphia County Medical Society

**Published:** 1889-01

**Authors:** 


### *PHILADELPHIA COUNTY MEDICAL SOCIETY.*

### Stated Meeting, November 14, 1888.

Prof. Forbes in the chair.

Dr. Joseph Price read a paper entitled: "A Consideration of some Recent Work in Abdominal Surgery." (See original communication in December number, p. 220.)

DISCUSSION.

Dr. Theophilus Parvin: My remarks will be chiefly in reference to the treatment of extra-uterine gestation. Quite agreeing with the writer that the certain diagnosis of this condition in the early weeks is impossible, and that the great majority of cases are recognized only after the rupture of the gestation cyst, I must think that those instances in which early recognition was asserted were altogether exceptional, and the recognition only a conclusion of probability, or a fortunate guess.

But an extra-uterine gestation being known, the question of treatment immediately presents itself. Different answers to this question are given. What may be called the American method, because more employed in this country than in any other, owes its origin to Dr. J. G. Allen, of this city, who successfully employed the faradic current for the purpose of destroying the life of the fetus. One of the criticisms made upon this method is that the proof of the extra-uterine gestation fails, in that no product of conception is revealed, the *corpus delicti* cannot be found; there may be as many as two or three exceptions—that is, some time after fetal life has been destroyed, an abscess has communicated with the exterior, and parts of the fetus been discharged. Nevertheless, the question has been asked, whether, in the long list of cases in which electricity was employed with such unusual success, there were some in which the fact of pregnancy was not conclusively proved.

In regard to those few cases of asserted interstitial pregnancy in which the fetus entered the uterus, obedient to the electric stimulus, and then was expelled through the natural passages, I must confess to the least scepticism as to the correctness of the diagnosis in all; for such a uniformity of successful results, the fetus in all cases behaving so well, seems extraordinary. Is it not, at least, probable that, in some instances, the rupture of the cyst would be into the abdominal, instead of, invariably, into the uterine cavity?

The injection of morphia into the fruit-sac, for the purpose of destroying the life of the fetus, is a method regarded with favor by some eminent German authorities. Even if always successful and devoid of danger, the same theoretical objection, which has been made to the treatment by electricity, applies to it. There are still other objections to both methods.

There remains the treatment by abdominal section. Now, this is applicable to cases of ectopic gestation, whether rupture has occurred or not, though in the former, it seems to me, it is imperative. Others beside Mr. Tait have had valuable experience in the surgical treatment of this affection, though none, probably, a tithe of his; thus Worth has operated seven times, with six recoveries, and so firmly convinced is he of the importance of abdominal section that he declares an extra-uterine gestation ought to be treated as a malignant tumor—that is, extirpated at the earliest moment.

At the Philadelphia Hospital, quite recently, the abdomen of a woman was opened on account of rupture of a gestation cyst; a large amount of clotted blood was found in the abdominal cavity, but no bleeding points discovered, and, therefore, no ligation of vessels was done, or extirpation of the fragments of the cyst; the woman's chances for recovery were vastly increased by the thorough cleansing of the abdominal cavity.

After having witnessed several operations for extra-uterine pregnancy performed with great skill, and the results being uniformly favorable, I am more and more convinced that this is the method of treatment for all cases, the only exceptions being an abdominal pregnancy so far advanced that there would be hope of extracting a living child at term (when the operation might be deferred until near the close of pregnancy), and an unruptured interstitial pregnancy.

A word as to tubal collections of pus in puerperal septicemia. I cannot believe this is frequent, either from the few post mortems of women dying of puerperal fever which I have seen, or from my reading; in the last edition of Schroeder's *Obstetrics*, 1888, for example, it is stated that occasionally, or sometimes, such collections are found. I cannot, therefore, hope that any great diminution of the mortality of puerperal fever will come through removal of pus-filled tubes.

The brilliant results obtained by Mr. Tait, and many operators in this city whom I might name—the almost total exemption from mortality which their statistics show—must not mislead us, for there are dangers in abdominal sections, and patients may die shortly after a so-called successful operation. Thus, a little more than two months ago, in conversation with Dr. Lombe Atthill, of Dublin, he told me of a lady operated upon by a distinguished surgeon, and she perished from hemorrhage a few hours after.

The treatment of pelvic abscesses by abdominal section is, of course, a valuable addition to therapeutic means. But are all intrapelvic inflammations with suppuration amenable to this means? Given a case of inflammation adjacent to the uterus, the parts matted together making a resisting mass as large as the two fists, or larger, the patient suffering from peritonitis, and having fever, can the offending pus be safely reached through the opened abdomen?

Then, too, are there not other limits to the employment of abdominal section in diseases of women? I do not object to the removal of the tubes in cases of pyosalpinx, on the false ground that the woman is thus rendered sterile, for a tube so diseased can never have its functions restored—it is, hopelessly, remedilessly ruined. But what of the removal of the ovaries for pain, or for certain nervous disorders? Does such removal cure or even palliate in the majority of cases? Here is a question that demands careful and large investigation. Doubtless, some cases of so-called menstrual epilepsy are benefited by the operation, but it is doubtful whether many absolute cures result. It may be questioned, too, whether pain in the ovaries, the organs being otherwise normal—the so-called ovaralgia—demands their extirpation. I have seen a woman whose ovaries had been removed on account of pain; the suffering returned as severely as ever, and then the stump of each pedicle was taken away, but not the slightest benefit followed—a year after the last operation she was as bad as before the first. I have myself removed the coccyx for well-marked coccygodynia, and for a time the benefit was marked; and then came just as severe pain in the sacrum as there previously had been in the coccyx. Let us honestly and impartially look at both sides of the picture, see the dark as well as the light offered, and not be carried away by contemplating only the latter.

Dr. M. Price: I agree with Dr. Parvin and the writer that the diagnosis of extra-uterine pregnancy in the earlier period is simply a lucky guess. I must differ from Dr. Parvin, however, when he doubts the feasibility of operation in a pelvis full of a great mass of inflammatory thickening. No matter how great the mass, or how extensive the adhesions, unless malignant, it can certainly be removed. I have had no trouble in tearing away adhesions until the mass in the pelvis was reached, a diseased tube found, removed, and abscesses opened and drained. I have seen but one bad result, and that was from the deprivation of food and stimulus; the nurse absolutely robbing the patient of it—a fact I did not discover until too late. I have encountered hemorrhage from the tearing of adhesions but once, in which case it was controlled by three ligatures on the bowel itself. The cause of hemorrhage in most cases of abdominal section is imperfect ligature. The ligature slips, and the patient bleeds to death. In tearing adhesions from the broad ligament, I once ruptured a vessel as large as the radial artery. I had no trouble from this after it was properly secured in the pedicle. The button is sometimes cut too short; the ligature which is holding the uterus between the broad ligaments like a guy-rope cannot stand the strain, the pedicle slips out, and the cavity is flooded. Here is one advantage of the drainage-tube. It gives warning of such an accident. The nurse ought to be trained to recognize the warnings, so that the operator may be summoned without loss of time.

The question of antiseptics, in these operations, is an important one. I must protest against statements upon this floor that operators who fail to use chemical antiseptics should be held criminally responsible. I say they should never be used in the peritoneal cavity. They increase the risks, and never benefit the patient. Cleanliness and readiness for emergencies are the requisites for abdominal surgeons. Dr. Bantock, and Mr. Tait, since he has abandoned Listerism, have results fully as good as any operators in the world. Such statements must not be permitted. They may bring danger and trouble upon fellow-practitioners conscientiously striving to do the very best for their patients, and, therefore, rejecting antiseptic solutions, as dangerous in themselves, and as leading to dangerous neglect of cleanliness by a sense of false security.

Dr. John B. Roberts: I am one of those surgeons who believe that any person who undertakes surgical operations at this stage of the world's history assumes a grave responsibility, is guilty of a wrong to his patient, if he does not guide himself by modern teachings in regard to the prevention of septic accidents. At the same time, I think that Dr. Price, and others who think with him, are giving themselves unnecessary anxiety as to the force in jurisprudence of the expressions made upon this floor, and elsewhere, by surgeons who give voice to the modern theories of operators' responsibilities. The word antiseptic is misconstrued. It does not necessarily refer to chemical agencies. The point is, Shall we have the old septic surgery or the modern non-septic surgery? So that infection be excluded, it makes no difference whether we exclude sepsis by chemical agents, by heat, or by absolute cleanliness. Under the influence of the teachings of Dr. Price and his brother, and the results obtained by them and their pupils, I have resorted with confidence to distilled water in abdominal and pelvic work. But that is simply a substitution of heat as an antiseptic agent; and it is antiseptic surgery that Dr. Price employs, or aseptic surgery, if he prefers that term, when he takes scrupulous precautions to secure absolute cleanliness of hands and instruments and all the details of the operation. There is no necessity to quarrel about words. The fact is, that it is the consensus of opinion of the men of the day who have a right to express opinions upon this matter, that the surgeon is bound to protect his patients by those means in which he has greatest confidence against the risks of sepsis, and that any operator who neglects this is guilty of a crime; and it is well to have that distinctly stated here, and in all medical societies, until the whole body of the profession realize that it is a cardinal principal of surgery. As I said before, we do not and need not pin our faith to chemical agents, though I am among those who find use for chemical agencies, but we must insist upon non-sepsis, and then we will have the best possible results.

Dr. H. A. Kelly: Some of my growing experience has led me to differ from some of the details of procedure recommended. Above all, I do not think it imperative nor wise to operate upon pus containing tubes and ovaries as soon as discovered. These cases are with few exceptions essentially chronic in their course; I operated last spring upon a woman who had carried a pelvic abscess for nineteen years. The natural history of this disease is one of attacks of recurring localized peritonitis, during the attacks they are exceedingly prostrated, and the danger of operation increased. I know of no other cases which improve so much and are so amenable to treatment. With rest and the use of hot water we will, after a few days or a week or two, find the great mass of fresh inflammatory deposit gone, and are then able to make out the outlines of the diseased uterus and tubes which we now find movable, and we can proceed to operate under more favorable circumstances. Where rupture has occurred and the inflammation is general, delay is fatal. Opening a sac which points into the vagina, is in some cases far better and safer surgery than abdominal section. In a case which has been mistaken for typhoid fever, and in which an excellent gynecologist had clearly diagnosed pelvic abscess, but wisely declined abdominal section on account of her prostrated condition, I operated *per vaginam* in September. After determining by palpation the point of greatest fluctuation, I separated the anterior and posterior walls of the vagina by Simon's specula, and gently lifting the cervix, without making traction, burned a hole into Douglas's cul-de-sac, which was filled by the tumor, opening a pus sac containing more than a pint of pus, washed it thoroughly, drained, and douched daily. The patient made an excellent recovery, walking into my office this morning. She was too weak for abdominal section, and her life was thus saved.

Three years ago I was able, before rupture, to diagnose tubal pregnancy. I operated before rupture, and I have the fetus in my possession now. A pathognomonic sign, which we do not wish to wait for, is diminution while under observation, in the size of a cyst, presenting the other signs of extra-uterine fetation, due to absorption of the amniotic fluid. It only occurs after the death of the fetus.

I am not a warm advocate of electrolysis, but it is an absurd mistake for an English writer to think that in America the sac is punctured in the operation of electric feticide. The great difficulty with many cases put down on the lists as ruptured tubal pregnancies, is that sufficient evidence is not presented to show us that the cases actually were pregnancies. Where the fetus is not found, we want more than doubtful microscopic signs.

Among the recent advances in abdominal surgery, I would call attention to an operation which I have devised to avoid the dangers of sepsis and hemorrhage, and the dangers and annoyances of the extra-peritoneal clamp method of treating the stump in supra-pubic hysterectomy.

I liberate and deliver the tumor with the uterus, and constrict the pedicle with a rubber tube, then trim off the tumor above the tube, leaving a cupped stump. This I very carefully bring together by a continuous buried suture, beginning at the bottom, which runs to and fro on the stump until it is closed, so that the top of the stump now looks like the mouth of a purse. Then, raising this, I pass a stout ligature deep into the uterine tissue on either side below the rubber tube with a sweep of my needle, and by tying this ligate to the uterine artery; then I cut the constricting tube, and if there is any hemorrhage from the lips of the sealed canal, I pass another deep ligature on either side, which controls all oozing. The abdominal cavity is now completely closed by stitching the peritoneum of the wall to the peritoneum of the stump, *above* the ligatures on the uterine artery, and leaving the sutures, which thus unite the two peritoneal surfaces, long. A gauze dressing is put over the whole. These ligatures are brought through a hole in the gauze, and clamped in a pair of ordinary long-bite dressing forceps, effectually preventing dragging and inversion. These sutures can be cut in seven to nine days. The result is perfect. My friend, Dr. Polk, tells me he has a plan in its essentials very similar to this.

Dr. J. M. Baldy: I quite agree with Dr. Parvin that it is a happy guess if we diagnosticate tubal pregnancy before rupture. In a case seen a year or more ago, all the signs which we would expect in a case of extra-uterine pregnancy were found present, and a diagnosis made in accordance with these facts. An ovarian cyst was found at the operation. It is claimed that such a mistake would not take place if due care were used. But such a well-known authority as Mann, of Buffalo, has made such a mistake; he treated his patient with electricity, killed the fetus, and later the case was operated on by Wylie, of New York, and no signs of extra-uterine pregnancy found. Dr. Kelly speaks of a shrinkage of the sac from absorption of the amniotic fluid being a pathognomonic sign of this disease. I have never heard of this being advanced as a sign by any one else, nor can I conceive of its occurring.

Puncture, as a treatment, can only be mentioned to be condemned. Electricity has the advantage of being able to kill the fetus and of saving the woman from the horrors of a severe surgical operation. It, however, has its disadvantages ; a mass is always left behind which will be likely to cause all the dangers that any other pelvic disease may ; it often ulcerates out, and it leaves the patient as much unsexed as the operation would. I think the gentlemen who remove other pelvic troubles with the knife and leave this one, are more than inconsistent. Again, rupture of some of the vessels in the sac wall may take place. Mann thinks that these dangers should not be taken into account, but as they form together quite a large per cent. of the total number of extra-uterine pregnancies, what sane man dare disregard them ? The electrical treatment has its positive and immediate dangers. Jauvrin has lost a patient by rupture of a blood-vessel, after killing the fetus. An electrical current passed through some pelvic growths always makes the patient worse. I have seen this happen in the hands of an experienced electrician, the patient being worse after every treatment. With the knife, no case has ever been killed, and when the operation is over no subsequent trouble can follow. The trouble can always be removed in the early periods. As soon as a probable diagnosis is made, a surgical operation should always follow.

In regard to operations for abscesses, I do not share Dr. Parvin's views ; I think these large adherent masses can always be removed without danger, and that such should be their treatment. After once beginning the operation, I should much more fear leaving it, than removing at any cost ; it is the incomplete operation which gives us the worst results. On the other hand, I most heartily agree with Dr. Parvin, that only *diseased* organs should be taken away. If the operation for vague pain, epilepsy, insanity, and nervous diseases, has any place, it is only after the most careful consideration and consultation, and in the most conservative hands.

With regard to the fibroid tumors, I think with Dr. Kelly that the extra-peritoneal method of treating the stump is a long and disagreeable one, on account of the sloughing. The intra-peritoneal method, which I had the pleasure of seeing Martin do several times in Berlin, is in every way preferable, if we can do it with equal safety. Although my cases treated extra-peritoneally have gotten well, I see no reason why those done by the other way should not also, and I shall be tempted to try it at the first opportunity. The method Dr. Kelly proposes is a half-way one, and loses some of the advantages of both the others, without gaining very much.

And now, Mr. President, one word in regard to antiseptics, since the subject has been brought forward so prominently again. My convictions on this subject are very strong, and are the result of much and very earnest hard study. I believe most firmly that germicidal agents used in the abdominal cavity are not only useless but most positively harmful. At all events, this subject is not to be considered closed ; it is open to discussion and trial, and I most earnestly protest against any such sweeping statements as have been made on this floor by Dr. Gross in times past, going before the world as the final dictum of this Society. Personally I never use chemical agents in my surgery, and I have the best of results. There are a number of other gentlemen in this city who follow the same practice. I will pick five or six such men and compare their results with those of any other six operators in Philadelphia, and if our results do not equal or better those of our opponents, I will concede the point. In view of these facts, Dr. Gross and others have no right, by any such statements as they have made, to put us in the position to be taken into court in a malpractice suit: this is exactly what such absurd statements will lead to. If a surgeon goes to an operation with dirty hands, an eighth of an inch of dirt under his finger-nails, dirty instruments and what not, because, forsooth, he has dipped his hands and instruments into a solution of carbolic acid or corrosive sublimate, he is to be exempt from responsibility; but those of us who have probably spent days carefully preparing for an operation, studying every detail and taking every rational precaution, because we do not choose to follow this absolute dictum of our wise masters, must be held responsible. Does any sensible man think that these solutions really penetrate the dirt under some operators' finger-nails and disinfect them? For my own personal safety sake, Mr. President, I must protest against the assumption of these men.

Dr. George E. Shoemaker: In regard to the diagnosis of extra-uterine pregnancy before rupture, the remark of Mr. Tait quoted by the writer is often referred to, but is not of as great weight as might at first appear. Mr. Tait has not said that he has failed to recognize a case, but that he has not seen one. One difficulty is this: Mr. Tait, for example, is an operator, not a man in general practice, and would be likely to see only cases brought him by others. These cases often occur in women previously healthy; their early symptoms are not very striking; therefore, they are not in the hands of the general practitioner, and are not brought to the notice of the expert diagnostician. The latter, then, is not at fault. A case was recently reported in the *Medical News*, in which the diagnosis was made before rupture, and in which operation proved it correct. I believe that if the general practitioner called an expert consultation early, and carefully chose the expert, the true nature of the case would be recognized in a far larger proportion of instances.

Dr. M. Price: Dr. Kelly's treatment of the pedicle is no more intra-peritoneal than if the wire clamp was used, and not half as safe. The ligature to pull up the stump in case of need, is an additional objection.

Dr. J. Price had withdrawn from the meeting before the above discussion took place, and had no opportunity of closing it.

